# Moderate-to-vigorous physical activity as a mediator between sedentary behavior and cardiometabolic risk in Spanish healthy adults: a mediation analysis

**DOI:** 10.1186/s12966-015-0244-y

**Published:** 2015-06-20

**Authors:** Antonio García-Hermoso, Vicente Martínez-Vizcaíno, Mairena Sánchez-López, Jose I. Recio-Rodriguez, Manuel A. Gómez-Marcos, Luis García-Ortiz, for the EVIDENT Group

**Affiliations:** Laboratorio de Ciencias de la Actividad Física, el Deporte y la Salud, Facultad de Ciencias Médicas, Universidad de Santiago de Chile, USACH, Santiago, Chile; Health and Social Research Center, Universidad de Castilla-La Mancha, Edificio Melchor Cano, Centro de Estudios Socio-Sanitarios, Santa Teresa Jornet s/n, 16071, Cuenca, Spain; Universidad Autónoma de Chile, Santiago, Chile; School of Education, Universidad de Castilla-La Mancha, Ciudad Real, Spain; The Alamedilla Health Center, Castilla y León Health Service–SACYL, USAL, IBSAL, Salamanca, Spain

**Keywords:** Sedentary lifestyle, Moderate-to-vigorous physical activity, Mediation analysis, Insulin resistance

## Abstract

**Background:**

Public health strategies for cardiovascular prevention highlight the importance of physical activity, but do not consider the additional potentially harmful effects of sedentary behavior. This study was conducted between 2010 and 2012 and analyzed between 2013 and 2014. The aim of the study was to analyze the relationship between sedentary behavior and cardiometabolic risk factors in the Spanish adult population and to examine whether this relationship is mediated by moderate-to-vigorous physical activity (MVPA).

**Methods:**

The cross-sectional study included 1122 healthy subjects belonging to the EVIDENT study. Sedentary behavior was objectively measured over 7 days using Actigraph accelerometers. We assessed waist circumference (WC), triglycerides-to-HDL-C ratio (TG/HDL-C), and mean arterial pressure (MAP), and undertook homeostasis model assessment (HOMA-IR). Linear regression models were fitted according to Baron and Kenny procedures for mediation analysis.

**Results:**

TG/HDL-C and HOMA-IR were significantly higher in adults who spent more minutes in sedentary activities after adjusting for potential covariates. However when MVPA was added to the ANCOVA models as covariate the effect of sedentary time on HOMA-IR disappeared. In addition, MVPA acted as a full mediator of the relationship between sedentary time and HOMA-IR. In contrast, subjects with lower levels of MVPA presented worse cardiometabolic profiles than those from higher MVPA categories, even after controlling for sedentary time and other potential confounders.

**Conclusions:**

These results suggest that both MVPA and sedentary time should be considered when developing cardiometabolic risk guidelines.

**Trial registration:**

NCT01083082.

## Background

Epidemiological studies have consistently shown that higher levels of light [1–3] and moderate-to-vigorous physical activity (MVPA) are related to lower prevalence and incidence of several chronic diseases, including metabolic and cardiovascular disease [4, 5].

Sedentary behavior is defined as any waking behavior characterized by energy expenditure less than or equal to 1.5 metabolic equivalents while in a sitting or reclining posture [6]. The association between sedentary behavior and cardiometabolic risk factors remains controversial. Thus, while some prospective studies have suggested that the time spent in sedentary behavior predicts higher levels of fasting insulin [7] and other cardiometabolic risk factors [8], independent of the amount of time spent in MVPA [8–10], other studies have found that the association between sedentary time and cardiovascular risk factor levels was attenuated [11] or disappeared [5, 12] after adjusting for time spent in MVPA. In addition, some studies have not succeeded in establishing an association between higher sedentary time and cardiometabolic health [13]. Therefore, it is important to clarify whether the relationship between sedentary behavior and cardiometabolic risk persists after adjusting for physical activity (PA) levels. A recent cross-sectional study suggests that sedentary behavior may not have health effects independent of PA (total daily accelerometer counts) [14]. However, current international public health strategies for cardiovascular prevention highlight the importance of MVPA [15]. Therefore, it seems necessary to clarify the mediating role of MVPA on the potentially harmful effects of sedentary behavior.

Mediation analysis is a statistical method that can be used to elucidate the processes underlying an association between two variables and the extent to which the association can be modified, mediated, or confounded by a third variable [16]. A mediation effect exists when a third variable (the mediator) is responsible for the influence of a given independent variable on a given dependent variable. The aim of this study was twofold: first, to examine the relationship between sedentary behavior and cardiometabolic risk factors in the Spanish adult population, and second, to clarify whether this relationship is mediated by MVPA.

## Methods

### Study design

This was a cross-sectional analysis of baseline data from EVIDENT study. The protocol of the EVIDENT study (NCT01083082) has been published elsewhere [17]. This study was conducted between 2009 and 2012 and analyzed in 2013 and 2014. The EVIDENT study aimed to analyze the relationship of PA and dietary pattern to the circadian pattern of blood pressure, central and peripheral blood pressure, pulse wave velocity, carotid IMT, and biological markers of endothelial dysfunction in active and sedentary individuals without arteriosclerotic disease.

### Study population

Subjects aged 20–80 years were selected through random sampling from offices of general practitioners from six health centers, each located in a different city from Spain. The exclusion criteria were the following: known coronary or cerebrovascular atherosclerotic disease, heart failure, moderate or severe chronic obstructive pulmonary disease, walking-limiting musculoskeletal disease, advanced respiratory, renal or hepatic disease; severe mental disease; treated oncological disease diagnosed in the past 5 years; status as a terminal patient, and pregnancy. These criteria were confirmed by the general practitioner based on the electronic clinical records and the information reported by for the subjects. From the 1553 subjects included in the EVIDENT study, 431 were excluded because they did not have measurements of any of cardiometabolic risk factors (277) or accelerometer data (154); therefore, the sample finally included 1122 subjects for the analysis. The study was approved by an independent ethics committee of Salamanca University Hospital (Spain) and of other center involved in the study, and all participants gave written informed consent according to the general recommendations of the Declaration of Helsinki [18].

### Measurements

The detailed description about how the clinical data were collected, the anthropometric measurements were made, and the analytical parameters were obtained has been published elsewhere [17].

#### Anthropometric measurements

Body weight was determined on two occasions using a homologated electronic scale (Seca 770) following calibration (precision ± 0.1 kg), with the patient wearing light clothing and no shoes. Height in turn was measured with a portable system (SECA 222), recording the average of two readings. Body mass index (BMI) and waist circumference (WC) were measured. The readings (in centimetres) of WC were taken at the end of a normal breath.

#### Resting blood pressure

Blood pressure taken in the clinical setting involved three measurements of systolic blood pressure (SBP) and diastolic blood pressure (DBP) using the average of the last two with a validated OMRON model M7 sphygmomanometer (Omron Health Care, Kyoto, Japan) and following the recommendations of the European Society of Hypertension [19]. Then, the mean arterial pressure (MAP) was calculated using the following formula: DBP + [0.333× (SBP – DBP)]. Also, antihypertensive drugs use was recorded. The anthropometric variables and blood pressure were measured by trained nurses.

#### Biochemical determinations

Venous blood sampling was performed between 08:00 and 09:00 h, after the individuals had fasted and abstained from smoking and the consumption of alcohol and caffeinated beverages for the previous 12 h. Several blood biochemical parameters were determined including lipoproteins, glucose, and insulin. The insulin sensitivity was determined by HOMA-IR (homeostasis model assessment of insulin resistance): fasting glucose level (mmol/L) × fasting insulin level (mU/ml)/22.5. Lipid-lowering drugs use was recorded. Medication information was collected from electronic medical records and it was confirmed in the interviews with participants.

#### Physical activity and sedentary behavior

Physical activity (PA) and sedentary behavior were measured by an ActiGraph GT3X accelerometer (ActiGraph, Shalimar, FL, USA) which has been previously validated [20, 21]. The GT3X accelerometer measures acceleration in three individual orthogonal planes (vertical, anteroposterior, and medio-lateral) and provides activity counts as a composite vector magnitude of these three axes.

Participants wore the accelerometer fastened with an elastic band to the right side of the waist for seven consecutive days with habitual PA. All subjects were verbally instructed on how to use the accelerometer. The participants were instructed to wear the accelerometer throughout the day from the time they woke up in the morning until they went to bed at night, except for bathing and performing activities in the water. Wear time was determined by subtracting non-wear time from 24 h. Non-wear time was defined by an interval of at least 60 consecutive min of zero activity counts, with allowance of up to 2 min of counts between 0 and 100. For the analyses, a valid day was defined as accumulating at least 600 min wearing the accelerometer. Intensity of PA was categorized according to the cut-off points proposed by Troiano et al. [22]: sedentary (<100 counts min), light (100–2019 counts min), moderate (2020–5998 counts min), and vigorous (>5999 counts min). MVPA time was calculated as the mean daily minutes ≥ 2020 counts per min from all valid days.

#### Behaviors

Smoking history was assessed through questions on smoking status (current smokers or nonsmokers) and alcohol consumption status (currently drinks or not drink alcohol beverages).

### Statistical analysis

Continuous variables were expressed as the mean ± SE for normally distributed continuous data, the median (interquartile range, IQR) for asymmetrically distributed continuous data. Categorical variables we expressed as n (%). Statistical normality of the variables was tested using both graphical (normal probability plot) and statistical procedures (Kolmogorov–Smirnov test). Due to their skewed distribution the following variables were log-transformed prior to analyses: BMI, WC, triglycerides, HDL-C, triglycerides-to-HDL-C ratio, and HOMA-IR. To aid interpretation, data were back-transformed from the log scale for presentation in the results. Also, we determined to perform the analyses by sex because it has been extensively described that adults men and women have differences in both PA and cardiometabolic patterns.

ANCOVA models were estimated to test the differences in cardiometabolic risk parameters by categories of sedentary time and MVPA establishing three categories (low = Q1; medium = Q2-Q3; high = Q4), and adjusting for age, smoking and drinking habit, and time accelerometer worn in a first step (model 1); adding MVPA in a second step or sedentary time when MVPA was the independent variable (model 2). When the outcome of interest was MAP or TG/HDL-C, we additionally adjusted for the use of antihypertensive or lipid lowering medication, respectively. Pairwise post-hoc comparisons were examined using Bonferroni test. Finally we test a sensitivity analysis by for different age groups (adult < 45 years; middle-aged: 45 to 65 years; and aged > 65 years).

To examine whether the association between sedentary time and cardiometabolic risk factors was mediated by MVPA, linear regression models were fitted using bootstrapped mediation procedures included in the PROCESS SPSS macro [23]. The first equation regressed the mediator (MVPA) on the independent variable (sedentary time). The second equation regressed the dependent variable (logBMI, logWC, logHDL-C, logTG, logTG/HDL-C ratio, PAM, and logHOMA-IR) on the independent variable. The third equation regressed the dependent variable on both the independent and the mediator variable.

The following criteria were used to establish mediation: (1) the independent variable is significantly related to the mediator; (2) the independent variable is significantly related to the dependent variable; (3) the mediator is significantly related to the dependent variable; and (4) the association between the independent and dependent variable is attenuated when the mediator is included in the regression model. The Sobel test was used to test hypothesis that the indirect effect was equal to zero. This analysis was adjusted by age, smoking habit, drinking habit, and time accelerometer worn.

Statistical analyses were performed with IBM SPSS 22.0 software, and the level of significance was set at α = 0.05.

## Results

Data were obtained from 1122 subjects (mean age 55.0 ± 13.6 years), 695 of whom were women. Table 1 displays subject characteristics, by sex. According to the consensus recommendation that states that adults should accumulate at least 30 min of daily MVPA, 45.3 % of participants could be considered active.

**Table 1:** Baseline demographic and clinical characteristics of subjects

	Total (n = 1122)	Men (n = 427)	Women (n = 695)	*p*
Age (years)	55.0 (0.4)	57.6 (0.5)	53.4 (0.4)	<0.001
Smoking status. n (%)				
Yes	224 (20.0)	79 (18.6)	144 (21.4)	<0.001
No or past	898 (80.0)	352 (81.4)	546 (78.6)	
Alcohol status. n (%)				
Yes	710 (63.2)	314 (73.5)	396 (57.0)	<0.001
No or past	412 (36.8)	113 (26.5)	299 (43.0)	
Weight (kg)	72.4 (0.4)	81.0 (0.5)	67.0 (0.4)	<0.001
Height (cm)	163.3 (0.2)	170.8 (0.3)	158.6 (0.2)	<0.001
Body mass index (kg/m^2^)	26.8 (24.1-29.8)	27.4 (25.6-30.1)	26.0 (22.9-29.5)	0.001
Waist circumference (cm)	92.0 (85.0-100.0)	97.0 (92.0-104.0)	88.0 (81.0-96.0)	<0.001
Triglycerides (mg/dL)	95.0 (71.0-131.0)	107.0 (80.5-152.2)	87.0 (66.0-121.0)	<0.001
HDL-cholesterol (mg/dL)	58.0 (48.0-68.0)	50.0 (43.0-59.0)	62.0 (53.0-72.0)	<0.001
TG/HDL-C (mg/dL)	1.6 (1.1-2.5)	2.0 (1.4-3.4)	1.4 (1.0-2.2)	<0.001
HOMA-IR	1.4 (0.8-2.2)	1.7 (0.9-2.5)	1.3 (0.8-2.1)	0.005
Lipid-lowering drugs, n (%)	187 (16.7)	98 (23.0)	89 (12.8)	<0.001
Office systolic blood pressure (mmHg)	125 (0.6)	130 (0.7)	121 (0.5)	<0.001
Office diastolic blood pressure (mmHg)	77 (0.3)	78 (0.4)	75.8 (0.3)	<0.001
Mean arterial blood pressure (mmHg)	91 (0.4)	96 (0.4)	88 (0.4)	<0.001
Antihypertensive drugs, n (%)	322 (28.7)	261 (61.1)	156 (22.4)	<0.001
Time accelerometer worn, min/day	931.2 (9.5)	941.6 (11.2)	924.8 (8.0)	0.239
Used valid days, n	5.5 (0.1)	5.4 (0.1)	5.6 (0.1)	0.994
MVPA, min/day	46.5 (1.1)	53.8 (1.5)	42.0 (0.9)	<0.001
Meet recommendations for MVPA^a^. n (%)	582 (51.9)	224 (52.4)	355 (51.1)	0.003
Sedentary time, min/day	580.3 (7.8)	601.7 (9.0)	567.2 (6.3)	0.003

*HDL* high-density lipoprotein, *TG* triglycerides, *MVPA* moderate-to-vigorous physical activity

Values are means (standard deviations (SE)) for normally distributed continuous data and medians (interquartile range (IQR)) for asymmetrically distributed continuous data and number and proportions (%) for categorical data; ^a^150 min/week of MVPA [14]

Mean differences in cardiometabolic risk parameters according to sedentary time categories are shown in Table 2. Subjects in the low sedentary time category had lower TG/HDL-C and HOMA-IR values than participants in the high category in model 1. Likewise, subjects in the medium sedentary time category accumulated lower TG/HDL-C ratio than the high category. Also, women in low category reported lower TG than high category. After adjusting for MVPA (model 2), the differences disappeared except for TG.

**Table 2:** Mean differences in cardiometabolic risk parameters by sedentary time categories controlling for potential confounders, by sex

	Crude data				Model 1				Model 2			
	Low (L)	Medium (M)	High (H)	*p*	Low (L)	Medium (M)	High (H)	* p*	Low (L)	Medium (M)	High (H)	*p*
BMI												
Men	28.0 ± 0.4	28.1 ± 0.3	28.2 ± 0.4	0.997	27.6 ± 0.6	28.1 ± 0.3	28.6 ± 0.5	0.968	27.9 ± 0.6	28.1 ± 0.3	28.1 ± 0.5	0.955
Women	26.4 ± 0.4	27.0 ± 0.3	27.2 ± 0.4	0.595	25.9 ± 0.5	26.9 ± 0.3	27.9 ± 0.5	0.215	26.5 ± 0.6	26.9 ± 0.3	27.3 ± 0.5	0.283
WC												
Men	98.1 ± 1.2	98.6 ± 0.8	98.9 ± 1.1	0.891	96.5 ± 1.5	98.6 ± 0.8	100.4 ± 1.4	0.985	97.2 ± 1.5	98.7 ± 0.8	99.5 ± 1.4	0.972
Women	89.3 ± 1.0	89.8 ± 0.7	90.3 ± 1.0	0.776	86.5 ± 1.2	89.5 ± 0.7	90.4 ± 1.2	0.496	87.8 ± 1.3	89.6 ± 0.7	92.1 ± 1.2	0.741
TG												
Men	131.3 ± 9.1	124.3 ± 6.0	124.3 ± 8.8	0.664	126.3 ± 11.9	124.7 ± 6.1	128.1 ± 10.8	0.755	127.2 ± 12.3	124.8 ± 6.2	127.8 ± 11.3	0.826
Women	92.4 ± 4.6	102.9 ± 3.1	103.3 ± 4.4	0.009	80.9 ± 5.6	102.5 ± 2.9	115.2 ± 5.1^a^	0.016	85.7 ± 5.8	102.6 ± 2.9	110.2 ± 5.3^a^	0.024
HDL-C												
Men	51.4 ± 1.4	51.5 ± 0.9	52.9 ± 1.3	0.571	53.3 ± 1.8	51.1 ± 0.9	51.9 ± 1.6	0.801	52.5 ± 1.8	51.0 ± 0.9	52.8 ± 1.7	0.832
Women	64.7 ± 1.3	62.5 ± 0.8	63.4 ± 1.2	0.688	66.5 ± 1.6	64.3 ± 0.8	65.3 ± 1.5	0.505	66.2 ± 1.7	62.2 ± 0.8	62.7 ± 1.6	0.433
TG/HDL-C ratio												
Men	2.6 ± 0.2	2.7 ± 0.2	2.9 ± 0.2	0.020	2.0 ± 0.3	2.6 ± 0.2^b^	2.8 ± 0.3^a^	0.022	2.7 ± 0.4	2.6 ± 0.1	1.7 ± 0.3	0.800
Women	1.6 ± 0.1	1.8 ± 0.1	2.2 ± 0.1	0.015	1.3 ± 0.1	1.8 ± 0.1^b^	2.1 ± 0.1^a^	0.035	1.4 ± 0.1	1.8 ± 0.1	1.9 ± 0.1	0.433
HOMA-IR												
Men	1.5 ± 0.1	1.8 ± 0.1	2.0 ± 0.2	<0.001	1.2 ± 0.2	2.0 ± 0.1	2.1 ± 0.2^a^	0.008	1.3 ± 0.2	1.6 ± 0.1	1.8 ± 0.2	0.168
Women	1.3 ± 0.1	1.7 ± 0.1	1.9 ± 0.1	0.005	1.0 ± 0.2	1.7 ± 0.1	2.2 ± 0.1^a^	0.017	1.5 ± 0.2	1.7 ± 0.1	2.0 ± 0.2	0.318
MAP												
Men	98.0 ± 1.3	97.0 ± 0.8	96.0 ± 1.2	0.636	98.9 ± 1.7	96.8 ± 0.9	95.4 ± 1.5	0.720	98.7 ± 1.7	96.8 ± 0.9	95.7 ± 1.6	0.725
Women	89.6 ± 1.0	91.3 ± 0.7	91.0 ± 1.0	0.102	89.4 ± 1.3	90.8 ± 0.9	92.1 ± 1.2	0.076	89.9 ± 1.4	90.9 ± 0.7	91.5 ± 1.2	0.083

Values are means ± SE. *HDL* high-density lipoprotein, *MAP* mean arterial pressure, *TG* triglycerides, *WC* waist circumference

Model 1: adjusted for age, smoking habit, drinking habit, and time accelerometer worn; TG, HDL-C and TG-HDL-C ratio was additionally adjusted for the use of lipid-lowering drugs (yes/no); MAP was additionally adjusted for the use of antihypertensive drugs (yes/no). Model 2: model 1 covariates + moderate-to-vigorous physical activity (mean min/day). ^a^ L < H; ^b^ M < H

Mean differences in cardiometabolic risk parameters according to MVPA categories are shown in Table 3. Participants in the low MVPA category had higher mean BMI, WC, and HOMA-IR values than participants in the high category in model 1, even after adjusting for sedentary time (model 2). Likewise, men in the low sedentary time category had higher BMI than men in the low-medium category. Equally, women classified with low MVPA showed higher values of WC, TG, HDL-C, and TG-HDL-C ratio, even after adjusting for sedentary time (model 2). Equally, sensitivity analysis by age categories showed similar results than whole sample (data not shown).

**Table 3:** Mean differences in cardiometabolic risk parameters by MVPA categories controlling for potential confounders, by sex

	Crude data				Model 1				Model 2			
	Low (L)	Medium (M)	High (H)	*p*	Low (L)	Medium (M)	High (H)	*p*	Low (L)	Medium (M)	High (H)	*p*
BMI												
Men	29.5 ± 0.4	27.6 ± 0.3	27.6 ± 0.4	<0.001	29.6 ± 0.4	27.6 ± 0.3^b^	27.5 ± 0.4^a^	<0.001	29.2 ± 0.7	27.5 ± 0.3^b^	28.0 ± 0.8^a^	0.001
Women	28.3 ± 0.4	26.7 ± 0.3	26.1 ± 0.4	0.008	28.3 ± 0.4	26.7 ± 0.3	26.0 ± 0.4^a^	0.005	27.1 ± 0.6	27.0 ± 0.7	26.6 ± 0.3^a^	0.005
WC												
Men	101.8 ± 1.1	99.2 ± 0.8	97.8 ± 1.1	0.002	102.1 ± 1.1	97.2 ± 0.8	93.6 ± 1.1^a^	0.002	101.3 ± 1.8	97.1 ± 0.8	98.5 ± 2.1^a^	0.002
Women	93.2 ± 1.0	89.5 ± 0.7	87.0 ± 0.9	0.001	92.7 ± 1.0	89.7 ± 0.7	87.1 ± 0.9^a^	<0.001	90.8 ± 1.5	89.8 ± 1.7	89.4 ± 0.7^a^	<0.001
TG												
Men	131.9 ± 8.6	122.6 ± 6.2	126.3 ± 8.7	0.660	129.8 ± 8.8	123.3 ± 6.3	125.1 ± 8.8	0.164	124.1 ± 14.5	122.7 ± 6.4	134.0 ± 16.5	0.181
Women	114.1 ± 4.5	99.7 ± 3.1	89.3 ± 4.4	0.002	113.2 ± 4.3	100.2 ± 3.0	89.2 ± 4.2^a^	<0.001	106.7 ± 6.7	99.3 ± 3.0	97.1 ± 7.5^a^	0.001
HDL-C												
Men	49.9 ± 1.3	51.5 ± 0.9	54.4 ± 1.3	0.056	50.5 ± 1.3	51.6 ± 0.9	53.7 ± 1.3	0.553	50.6 ± 2.1	51.6 ± 0.9	53.6 ± 2.5	0.554
Women	59.7 ± 1.2	63.4 ± 0.9	63.4 ± 1.2	0.002	59.8 ± 1.3	63.4 ± 0.8	66.3 ± 1.2^a^	0.005	60.3 ± 1.9	63.5 ± 0.9	65.6 ± 2.2^a^	0.005
TG/HDL-C ratio												
Men	2.9 ± 0.2	2.6 ± 0.1	2.6 ± 0.2	0.443	2.9 ± 0.2	2.6 ± 0.2	2.7 ± 0.2	0.336	2.7 ± 0.4	2.6 ± 0.2	2.9 ± 0.5	0.345
Women	2.1 ± 0.1	1.7 ± 0.1	1.5 ± 0.1	<0.001	2.1 ± 0.1	1.7 ± 0.1	1.5 ± 0.1^a^	<0.001	2.0 ± 0.2	1.7 ± 0.1	1.5 ± 0.2^a^	<0.001
HOMA-IR												
Men	2.3 ± 0.2	1.8 ± 0.1	1.5 ± 0.2	<0.001	2.4 ± 0.1	1.7 ± 0.1	1.4 ± 0.2^a^	0.002	2.3 ± 0.2	1.7 ± 0.1	1.5 ± 0.3^a^	0.002
Women	2.1 ± 0.1	1.6 ± 0.1	1.3 ± 0.1	0.001	2.1 ± 0.1	1.6 ± 0.1	1.3 ± 0.1^a^	<0.001	2.0 ± 0.2	1.7 ± 0.1	1.6 ± 0.2^a^	<0.001
MAP												
Men	96.8 ± 1.2	96.7 ± 0.9	97.6 ± 1.2	0.831	96.9 ± 1.3	96.8 ± 0.9	97.5 ± 1.3	0.785	98.7 ± 2.0	96.9 ± 0.9	95.3 ± 2.3	0.791
Women	91.0 ± 1.0	90.8 ± 0.7	90.6 ± 1.0	0.835	90.8 ± 1.0	90.9 ± 0.7	90.5 ± 0.9	0.666	90.1 ± 1.5	90.4 ± 0.1	95.0 ± 1.7	0.676

Values are means ± SE. *HDL* high-density lipoprotein, *MAP* mean arterial pressure, *TG* triglycerides, *WC* waist circumference

Model 1: adjusted for age, smoking habit, drinking habit, and time accelerometer worn; TG, HDL-C and TG-HDL-C ratio was additionally adjusted for the use of lipid-lowering drugs (yes/no); MAP was additionally adjusted for the use of antihypertensive drugs (yes/no). Model 2: model 1 covariates + moderate-to-vigorous physical activity (mean min/day). ^a^ L > H; ^b^ L > M

Finally, when we tested a first order interaction term between MVPA categories (active and non-active) with each cardiometabolic risk parameters we did not find statistical significance (p > 0.05), thus we assumed no moderation/effect modification in the mediation analysis.

### Mediation analysis

**Fig. 1:**
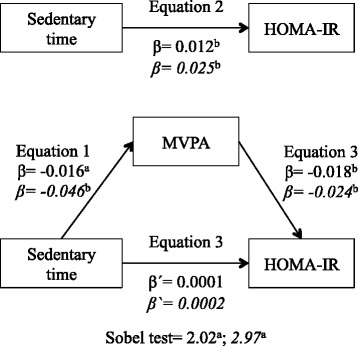
MVPA mediation model of the relationship between sedentary time and HOMA-IR, adjusting for potential confounders, by sex. Data in roman type refer to men. Data in italics refer to women. ^a^ *p* ≤ 0.05; ^b^ *p* ≤ 0.001

In both sexes, when we tested the mediator role of MVPA in the relationship between sedentary time and HOMA-IR, in the first regression equation sedentary time was negatively associated with MVPA. In the second equation, sedentary time was positively associated with HOMA-IR. Finally, in the third equation, when sedentary time and MVPA were simultaneously included in the model, MVPA was negatively associated with HOMA-IR (p ≤ 0.001) and although sedentary time remained positively associated with HOMA-IR, these associations did not maintain their statistical significance. These results suggest that the effect of sedentary time on insulin resistance was fully mediated by MVPA. Using the Sobel test for mediation it was estimated that in men 15.6 % (z = 2.02; p = 0.019) and in women 21.1 % (z = 2.97; p = 0.003) of the total effect of sedentary time on HOMA-IR was mediated by MVPA (Fig. 1).

Conversely, the relationship between sedentary time and BMI, WC, TG, HDL-C, TG/HDL-C ratio and MAP was not mediated by MVPA, since the above-mentioned criteria for the mediation analysis were not observed (data not shown). Equally, the relationship between MVPA and cardiometabolic risk parameters was not mediated by sedentary time.

## Discussion

Studies aiming to analyze the effect of sedentary behavior on cardiometabolic risk are scarce. Furthermore, it is unclear whether MVPA might act as a mediator in the relationship between sedentary behavior and cardiometabolic risk. The current study is, to our knowledge, the first aimed at analyzing whether PA acts as a mediator in the relationship between sedentary time and cardiometabolic risk. Data showed that sedentary time was positively associated with cardiometabolic risk parameters even after adjusting for socio-demographic and lifestyle potential confounders. Only the association between sedentary time and HOMA-IR became non-significant when we adjusted for MVPA, while the associations between sedentary time with WC, and TG/HDL-C ratio remained significant. Therefore, the data suggest: first, that the influence of sedentary time on obesity and lipid profile is independent of MVPA levels; second, the influence of sedentary time on insulin resistance is mediated by MVPA levels.

The relationship between sedentary behavior and cardiometabolic risk components has been reported in studies of young adults [3, 8], adults [5, 9], and older adults [10]. Our data suggest that subjects who spent more time in sedentary activities had worse cardiometabolic risk levels than those in other categories of sedentary time, even after adjusting for MVPA, except for HOMA-IR, where statistical significance disappeared after including MVPA in the model. Therefore, results suggest that, independent of MVPA levels, sedentary time is associated with a worsening of adiposity and lipid profile, which is consistent with other prospective studies conducted in the general population [3, 8–10]. This independence could be due to an increased energy intake, because watching television, a common sedentary behavior in the study population [24], is often accompanied by snacking and subconscious overconsumption [25]. Therefore, participants tend to have unhealthy dietary patterns, which may explain the positive association between sedentary time and abdominal obesity [26] and lipid profile [27], independent of MVPA. Finally, our non-significant findings for blood pressure are in agreement with the results from most previous studies [4, 28, 29].

As previously mentioned, although traditionally sedentary behavior has been considered as a predictor of cardiometabolic risk [9, 30–33], and the relationship between cardiometabolic parameters and MVPA has been extensively established [5, 12], it has not been fully clarified whether MVPA acts as a confounder or as a mediator. The mediation analysis confirms that the association between sedentary time and insulin resistance is fully mediated by MVPA. In this sense, data from several studies indicate a significant relationship between sedentary time and HOMA-IR in univariate analyses, however after statistically adjusting for MVPA these associations are no longer significant [4, 34, 35]. Thorp et al. [36] have suggested that the decrease in skeletal muscle contraction from sedentary behavior suppresses skeletal muscle glucose transporter type 4 (GLUT-4) and lipoprotein lipase activity, favoring an elevated level of plasma-free fatty acids, triglycerides, and glucose. Therefore, the role of MVPA as a mediator might be due to it: improving insulin-mediated glucose uptake; improving insulin action by increasing GLUT-4 expression in skeletal muscles [37, 38]; reducing blood glucose and the risk of insulin resistance [39] and; inducing alterations in fatty acid partitioning within the muscle cells in insulin sensitivity [40].

If that is the case, the present study provides new insights supporting the belief that for diabetes type 2 preventive interventions to be more effective, they should aim to achieve both an increase in MVPA and a reduction in sedentary behavior [8, 41]. In relation to this, a recent study found that the harmful effects of sedentary behavior may be largely mitigated through displacing time in other activities that require movement [42]. Therefore, the level of MVPA might also be a protective factor against the harmful effects of sedentary behavior on glucose metabolism. However, to date, the minimal amount of physical activity needed to prevent cardiometabolic risk is unknown.

Some limitations of this study should be acknowledged. First, the cross-sectional design prevents us from establishing a causal relationship. Second, because participants are conscious of wearing the accelerometer, an observer bias that might have influenced their daily PA cannot be discounted. Third, since participants belonged to a clinical trial including subjects randomly selected from out-patient clinics from different regions of Spain, the sample might not be representative of the general Spanish population. Fourth, the generalizability of the study could be compromised due to participants having met the rigorous inclusion criteria of the parent trial, in addition to the impossibility of adjusting for all potential confounders which cause the residual confounding to tease out independent associations. Fifth, our analysis was only focused on one component of the PA spectrum, so it is difficult to know whether the effects of sedentary behavior are indeed independent, or whether adults were benefitting from the light or total PA, but because of multi-collinearity problem this level of activity could not be included in ANCOVA models [14]. Finally, we have not included the dietary intake data as covariate, a parameter that could affect the observed results.

## Conclusions

In summary, the findings are significant from a clinical and public health point of view because they show that MVPA in adults is a mediator in the relationship between sedentary behavior and insulin resistance. Thus our data support that healthy lifestyle recommendations should encourage both the promotion of MVPA and the strategies to avoid sedentary behaviors in order to mitigate cardiometabolic risk.

## Abbreviations

BMI: Body mass index
BP: Blood pressure
HDL-C: High-density lipoprotein cholesterol
HOMA-IR: Homeostasis model assessment of insulin resistance
MAP: Mean arterial pressure
MVPA: Moderate-to-vigorous physical activity
TG: Triglycerides
WC: Waist circumference
